# Supplementary material for: BRAF Mutant Melanoma Adjusts to BRAF/MEK Inhibitors via Dependence on Increased Antioxidant SOD2 and Increased Reactive Oxygen Species Levels
Source: Cancers (Basel). 2020 Jun 23;12(6):1661. doi: 10.3390/cancers12061661 (PMC7352565; doi:10.3390/cancers12061661)
Supplement: Supplementary file 1 [file cancers-12-01661-s001.pdf]

Supplementary Material: BRAF mutant melanoma adjusts to BRAF/MEK inhibitors via dependence on increased antioxidant SOD2 and increased reactive oxygen species levels

Long Yuan, Rosalin Mishra, Hima Patel, Samar Alanazi, Xin Wei, Zhijun Ma, Joan T. Garrett

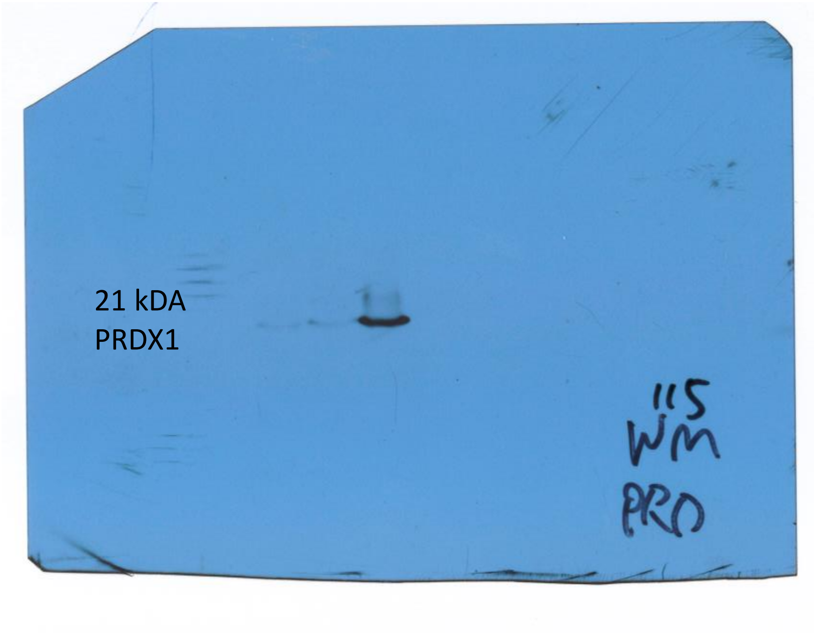

Figure S1. Whole blot image of PRDX1 in WM-115, WM-115 DR, WM-115 TDR from Figure 4.

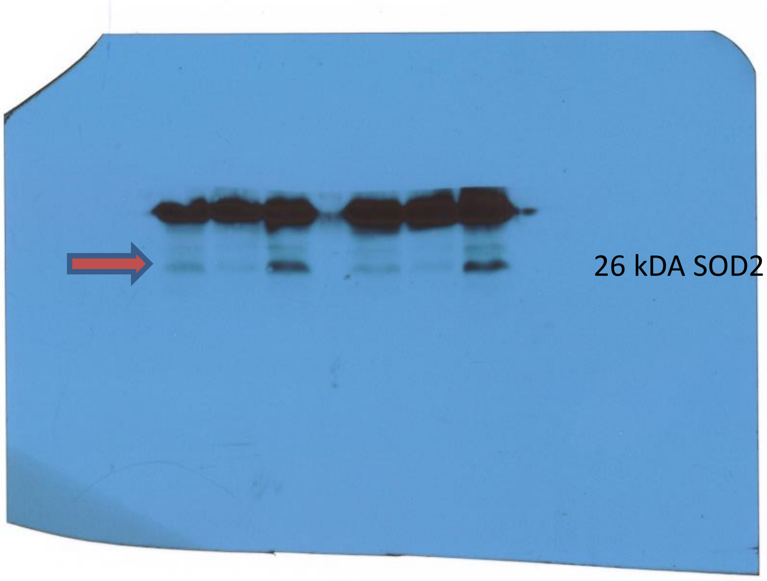

Figure S2. Whole blot image of SOD2 in WM-983, WM-983 DR, WM-983 TDR, WM-115, WM-115 DR, WM-115 TDR from Figure 4.

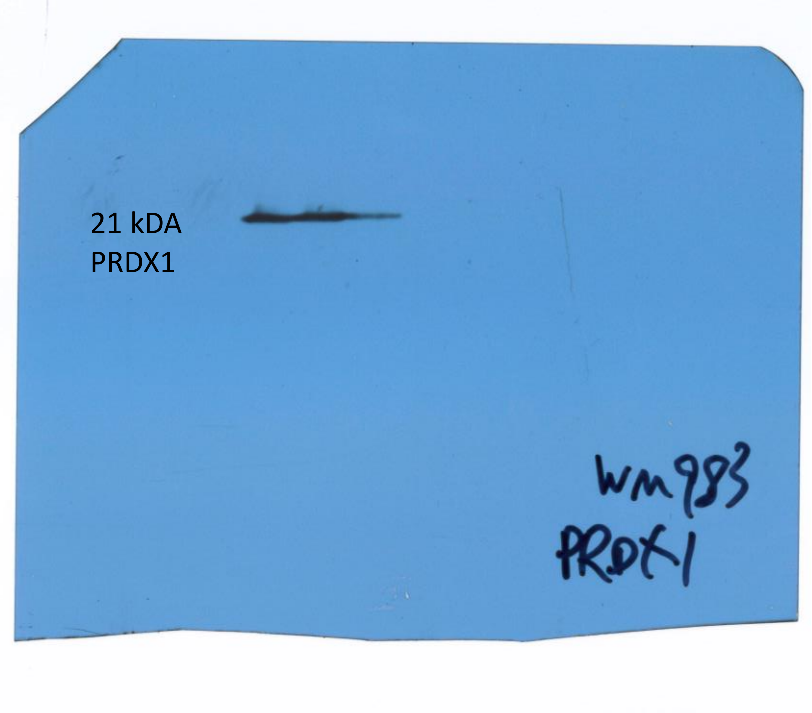

Figure S3. Whole blot image of PRDX1 in WM-983, WM-983 DR, WM-983 TDR from Figure 4.

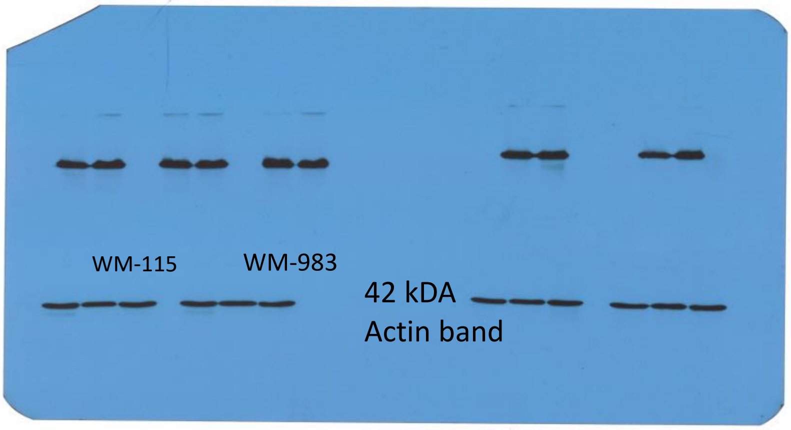

Figure S4. Whole blot image of Actin in WM-983, WM-983 DR, WM-983 TDR, WM-115, WM-115 DR, WM-115 TDR from Figure 4.

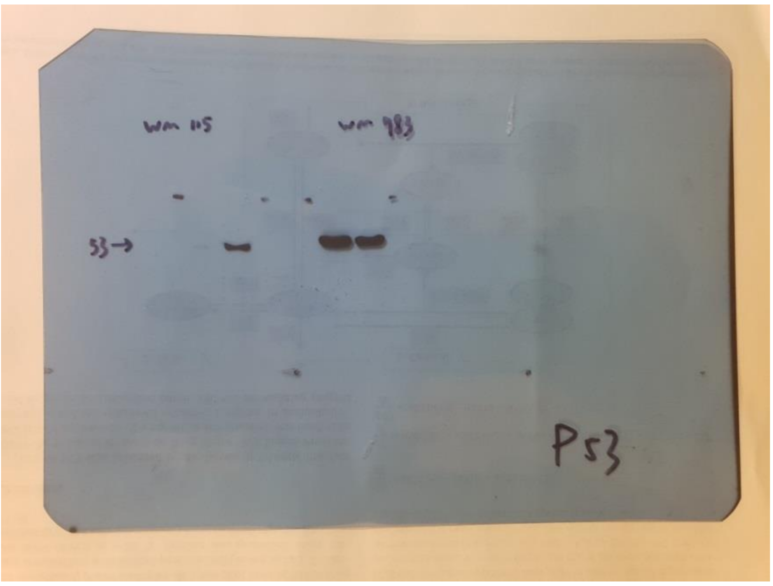

Figure S5. Whole blot image of p53 in WM-115, WM-115 TDR, WM-983, WM-983 TDR from Figure 4.

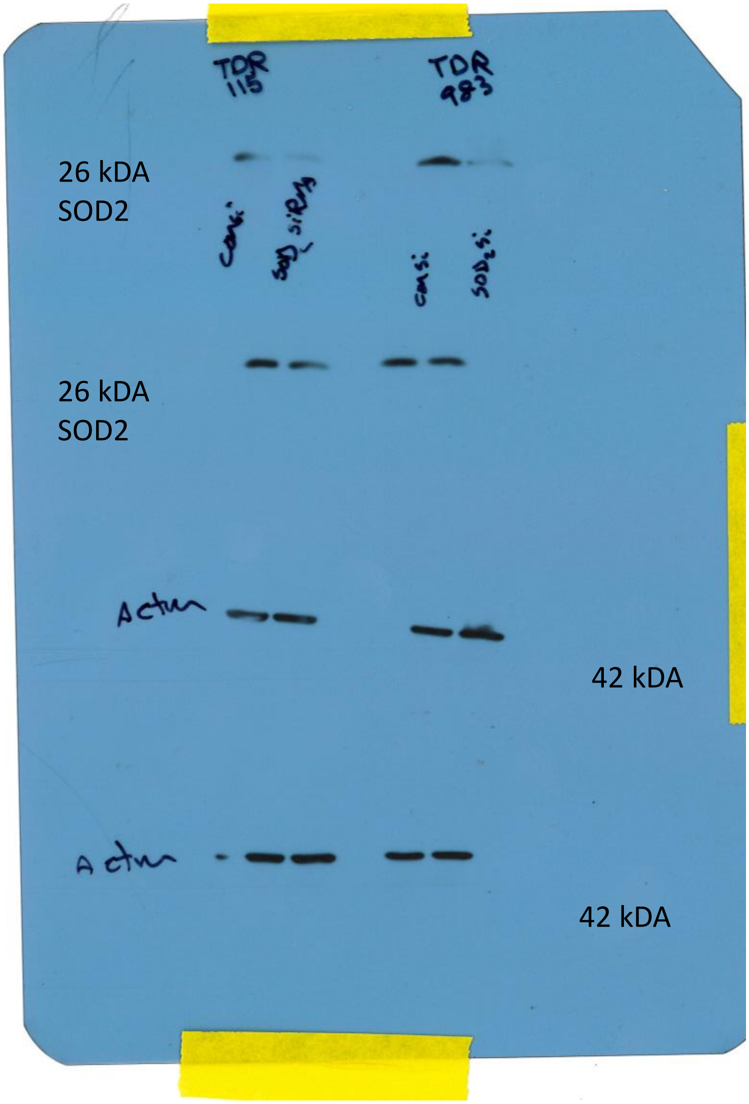

Figure S6. Whole blot image of Actin, CTRL SiRNA, SOD2 SiRNA in WM-983, WM-983 TDR, WM-115, WM-115 TDR from Figure 5.

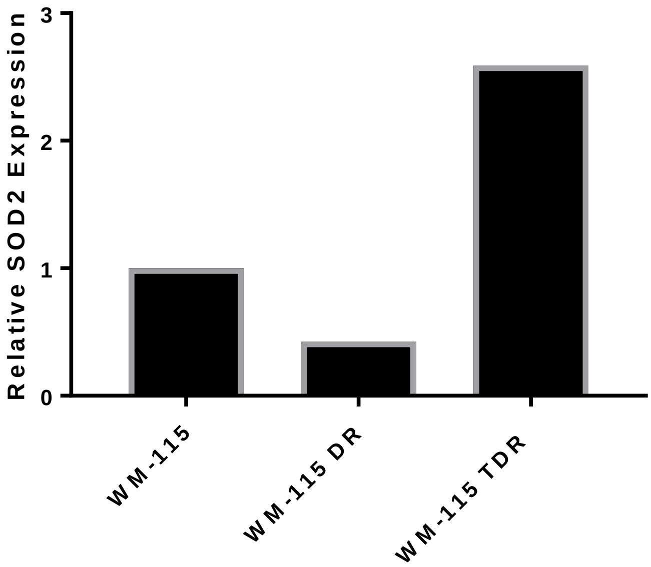

Figure S7. Densitometry reading of SOD2 expression in Figure 4A.

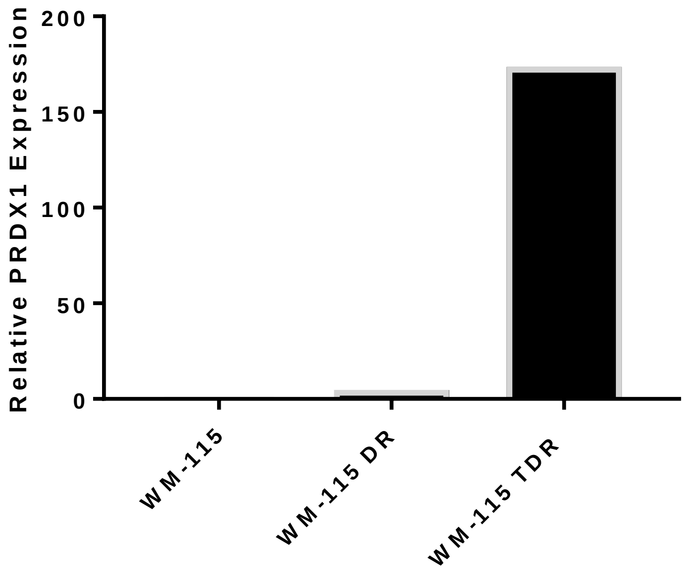

Figure S8. Densitometry reading of PRDX1 expression in Figure 4A.

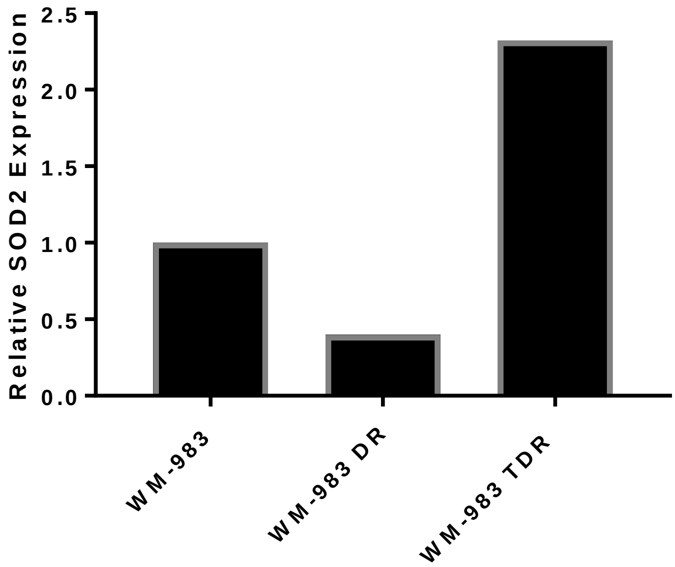

Figure S9. Densitometry reading of SOD2 expression in Figure 4B.

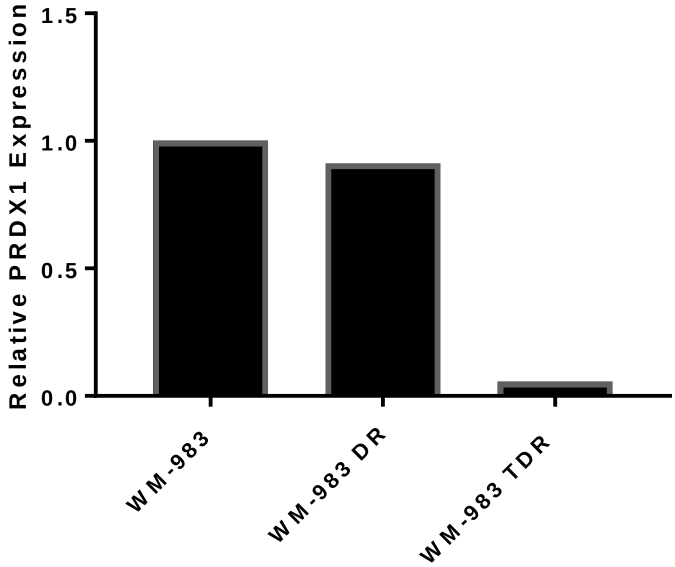

Figure S10. Densitometry reading of PRDX1 expression in Figure 4B.

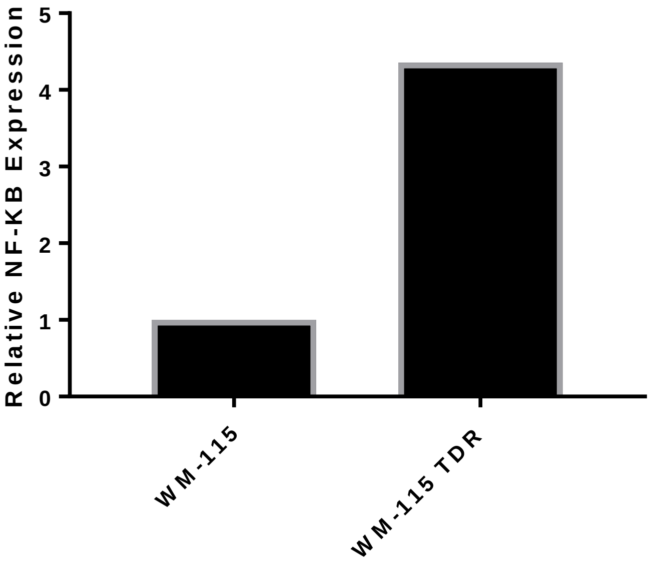

Figure S11. Densitometry reading of NF-KB expression in Figure 4C.

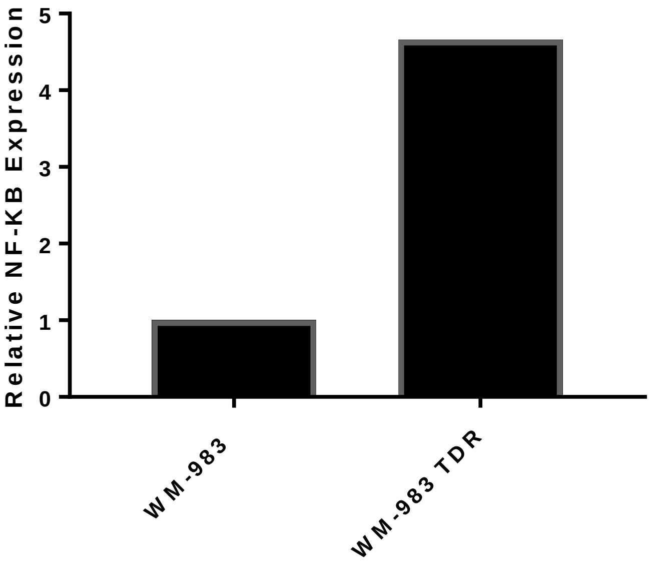

Figure S12. Densitometry reading of NF-KB expression in Figure 4C.

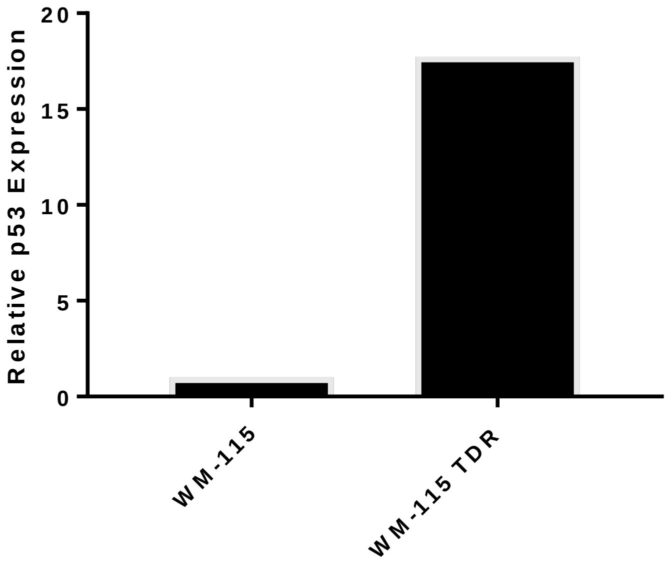

Figure S13. Densitometry reading of p53 expression in Figure 4C.

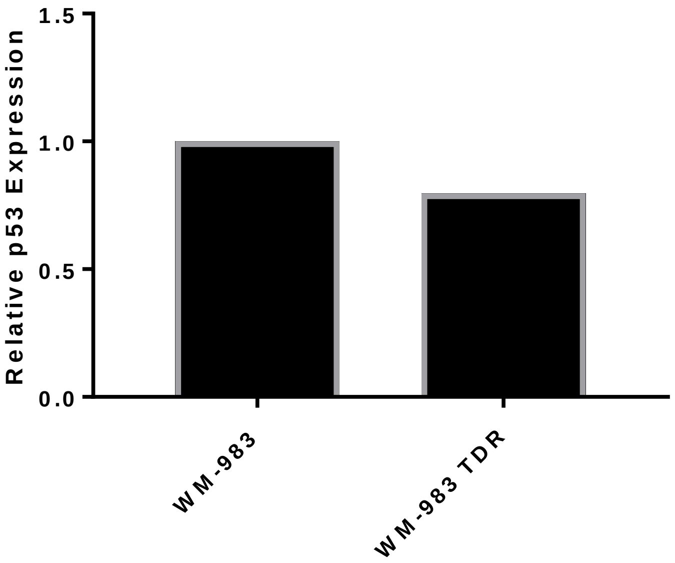

Figure S14. Densitometry reading of p53 expression in Figure 4C.
